# Physical fitness changes among school-aged children during the COVID-19 lockdown evaluated within the Hungarian National Student Fitness Test cohort

**DOI:** 10.1038/s41598-026-41055-8

**Published:** 2026-02-23

**Authors:** Ferenc Vincze, Tamás Csányi, Mónika Kaj, Katalin Kälbli, Gabriella Nagy-Pénzes, Tamás Pinczés, Alexandra Cselkó, János Sándor

**Affiliations:** 1 Department of Public Health and Epidemiology, Faculty of Medicine, University of Debrecen, Debrecen, Hungary; 2 Department of Physical Education Theory and Methodology, Hungarian University of Sports Science, Budapest, Hungary; 3 Hungarian School Sport Federation, Budapest, Hungary; 4 Bárczi Gusztáv Faculty of Special Needs Education, Eötvös Loránd University, Budapest, Hungary; 5 Department of Physical Education and Sport, Ferenc Kölcsey Teacher Training Institute, Debrecen Reformed Theological University, Debrecen, Hungary

**Keywords:** Adolescents, COVID-19, Fitness monitoring, Physical fitness, School, Health care, Medical research, Physiology, Risk factors

## Abstract

This cohort-based analysis aims to compare the health-related physical fitness levels of Hungarian youth before and one year after the first wave of the COVID-19 pandemic. We also aimed to analyze the impact of the school environment on students’ physical fitness. A cohort study was conducted to evaluate various physical fitness components, including body mass index (BMI), body fat percentage (BF%), cardiorespiratory fitness, musculoskeletal fitness, and flexibility, measured via the Hungarian National Student Fitness Test (NETFIT^®^). The analysis included 285,465 students across the pre-pandemic (2018/2019) and post-pandemic (2021/2022) academic years. Generalized linear mixed-effects models were used to quantify differences. The findings indicated a decline in BMI, BF%, cardiorespiratory fitness, flexibility, and musculoskeletal fitness measured with push-up and curl-up tests. However, standing broad jump, trunk-lift, and handgrip strength increased during the COVID-19 pandemic. Moreover, significant variations in students’ physical fitness across schools suggest that school-level factors play a crucial role in shaping students’ physical performance. This study highlights a general decline in adolescents’ physical fitness, likely linked to reduced physical activity due to COVID-19 pandemic related restrictions, which contributed to increased sedentary behavior and fewer outdoor activities. The extent of these changes varied across schools.

**Supplementary Information:**

The online version contains supplementary material available at 10.1038/s41598-026-41055-8.

## Introduction

Physical fitness refers to a set of attributes related to an individual’s ability to perform daily activities and is commonly assessed through measures of muscular strength, endurance, flexibility, body composition and cardiorespiratory capacity. Recent studies have demonstrated engaging sufficient physical fitness during childhood positively impacts physical, physiological, and psychological health and reduces the risks of various diseases, including blood pressure^1^ and adiposity^2^ while enhancing glycemic control^3^, bone health^4^, and cognitive function^5^.

Schools play crucial roles in fostering physical fitness by offering diverse opportunities, including daily physical education classes, extracurricular sports programs, and other structured activities that promote movement. In Hungary, Section  27 of Act CXC of 2011 on National Public Education mandates that schools provide five physical education classes per week, each lasting 45 min. Studies regarding the impact of legislation have confirmed the effectiveness of daily physical education in enhancing youth physical fitness, demonstrating significant improvements in various fitness components, such as cardiovascular endurance, muscular strength, and overall physical well-being^6^.

The COVID-19 pandemic has significantly disrupted daily life and raised particular concerns about children’s physical fitness. Notably, the beneficial effects of regular physical activity on physical health were attenuated during periods of COVID-19–related school closures^7^. Beyond these, children faced challenges such as social distancing, remote learning, and restricted access to outdoor spaces and recreational activities. The temporary closure of public places, sports facilities and educational institutions significantly limited opportunities for physical activity, which in turn likely contributed to declines in physical fitness by reducing engagement in activities that maintain muscular strength, endurance, and cardiovascular capacity. Research examining physical activity levels during the COVID-19 pandemic revealed concerning trends. The proportion of children meeting recommended physical activity guidelines decreased by nearly half^7^, while sedentary behaviour and screen time increased substantially^7–9^. Based on the literature, these changes in activity patterns were associated with declines in physical fitness, including reductions in muscular strength, endurance, and cardiovascular capacity^10–12^.

Despite substantial research efforts by multiple working groups, key gaps in knowledge persist. Most investigations rely on cross-sectional data^13–16^, target specific subgroups^17^, or assess only one aspect of physical fitness^18^ (such as body mass index changes or cardiovascular health). Additionally, many studies overlook broader socioeconomic and environmental factors that may influence physical fitness outcomes. However, certain non-individual factors—such as rural–urban school location, teacher characteristics (including confidence, motivation, and the value placed on physical fitness), and school-level resources (such as available space and facilities)—may also influence students’ physical fitness and could exacerbate the negative effects of school closures^19–21^.

These methodological problems are reflected in the debatable impact of the current COVID-19 pandemic or the preparedness for future pandemics. Realizing the weight of this challenge, a recent Delphi study of international experts ranked the top research priorities for children’s physical fitness, highlighting longitudinal studies regarding fitness‒health links, surveillance systems for decision-making, and standardized international fitness surveys^22^.

Regular school level based data collection system that can be adapted to these standards have been implemented in numerous countries and regions^23^, including in Hungary since 2015. The Hungarian National Student Fitness Test (NETFIT^®^) is an annual, mandatory physical fitness assessment system conducted in all schools. It evaluates the fitness levels of students in grades 5–12 using a criterion-based method aligned with health standards^24^. The annual assessment provides a unique opportunity to track population-level changes over time, including the impact of the COVID-19 pandemic. Because the system is nationwide and mandatory, it generates highly representative data, minimizes selection bias, and provides population-level estimates. In addition, the repeated annual collection allows for adjustment for school-level characteristics, enabling analyses that account for differences in school performance, resources, and contextual factors.

During the second semester of the 2019–2020 school year and the entire 2020–2021 academic year, schools were closed for approximately half of the total standard school days in Hungary^25^. In response to this crisis, educational institutions transitioned to remote learning models, including physical education classes, and organized sports activities were cancelled. During this period, teachers delivered online instruction through text and video materials, which formed the primary content of physical education lessons implemented in a distance learning format. The effectiveness of distance physical education lessons is questionable, as some students reported that they were not active at all, and in some schools, the number of classes was reduced or cancelled during the closures^26^.

Despite existing recommendations suggesting multiple strategic approaches (such as home-based exercise, dance, walking, and outdoor free-play) for young populations to keep active during the lockdown^27^, we hypothesized that physical fitness levels were reduced due to confinement. We further hypothesized that students’ residential location and school characteristics—type and maintainer, as proxies for school-level resources—may have influenced these changes unevenly. Therefore, this cohort-based analysis aims to compare the body composition cardiorespiratory, muscular, flexibility fitness of Hungarian youth in grades 5 to 8 before and after the first wave of the COVID-19 pandemic. We also aimed to analyze the impact of the school environment on students’ physical fitness.

## Methods

### Study design and participants

The data for this study were obtained from the NETFIT^®^ data collection system. The main objective of the NETFIT^®^ is to monitor and follow up the physical fitness of Hungarian school-aged (grades 5–12) children using a standardized data collection procedure. Full descriptions of the data collection system have been reported elsewhere^24^. In brief, the NETFIT^®^ system involves more than 3,700 schools, 700,000 students and 13,000 teachers every year, which constitute 100% of all public and private schools with all children above 5^th^ grade in the primary and secondary school population. The annually conducted tests in NETFIT^®^ are a compulsory and standardized fitness measurement method^24^ of the Hungarian public education system developed by the Hungarian School Sport Federation (HSSF) in cooperation with the Cooper Institute (Dallas, TX)^24^.

This study used data from the 2018/2019 (pre-pandemic) and 2021/2022 (post-pandemic) school years, which were the only periods with complete data collection. Assessments were suspended during the 2019/2020 and 2020/2021 school years due to COVID-19–related lockdowns; therefore, individual-level fitness measurements collected before and after the pandemic were compared. In the 2018/2019 school year, legislation permitted a 19-week assessment period starting on 1 January, whereas in 2021/2022 assessments were allowed throughout the entire school year (September to June).

In the reporting periods, approximately 90% (pre-pandemic: 88.9%, post-pandemic: 90.4%) of the targeted student numbers were recorded in the NETFIT^®^ IT system by the physical education teacher. This cohort study utilized data collected at two time points from the NETFIT^®^ program. The baseline cohort comprised students in grades 5 and 8 during the pre-pandemic school year, with those having eligible fitness data qualifying for inclusion in the final cohort. Follow-up data collected in the post-pandemic school year were linked to the baseline records at the individual level, enabling direct within-student comparisons between pre-pandemic and post-pandemic measurements. Of the original cohort, 11% of students were lost to follow-up, resulting in a follow-up rate of 89% (students lost to follow-up were excluded from the analyses).The study was approved and supervised by the scientific board of HSSF, which is responsible for both the utilization of NETFIT^®^ database and the protection of human rights in handling sensitive personal data (410IB23). The study was conducted in accordance with the Declaration of Helsinki.

### Definition of the measures

The NETFIT^®^ fitness measurement system distinguishes between four different fitness profiles (body composition, cardiorespiratory fitness (aerobic capacity), musculoskeletal fitness and flexibility profile), each of which has a different fitness test.

Prior to its nationwide implementation, the NETFIT^®^ system was preceded by a nationally representative youth fitness study in Hungary, which assessed the applicability of FITNESSGRAM^®^ test items and standards in Hungarian children and adolescents^24^. Based on this assessment, the original sex- and age-specific FITNESSGRAM^®^ cut-off values developed by the Cooper Institute were adopted^24,28–32^ without modification for most fitness components^29^. Standing long jump^32^ and handgrip strength^31^ were retained as traditional Hungarian test items; for these measures, nationally derived reference values were applied.

In accordance with The Cooper Institute’s^®^ Fitnessgram, fitness scores are classified into three zones (Cooper Institute, TX, USA): (1) the healthy fitness zone (HFZ), (2) the needs improvement zone (NIZ) and (3) the needs improvement: health risk zone (NIHR). These classifications are based on students’ age and sex (using sex- and age-specific cut-off values). In this study, two zones (NIZ and NIHR) were combined and referred to as the not meeting the healthy fitness zone (NMHFZ). The NETFIT^®^ system distinguishes an underweight category for body mass index and body fat percentage, with BMI classification based on the international cut-offs defined by Cole et al.^33^. In this analyses, respondents in this category were treated and categorized into the HFZ.

As part of the body composition profile, weight (kg) and height (cm) were measured according to standard procedures and used to calculate body mass index (BMI, kg/m^2^). Body fat percentage (%BF) was measured using the Omron BF511 device (OMRON Healthcare Co. Ltd., Japan), whereas aerobic capacity was measured through the administration of the progressive aerobic cardiovascular endurance test (PACER). Musculoskeletal fitness was assessed through several fitness tests, including the standing broad jump test, push-up test, curl-up test, trunk-lift test, and handgrip strength test. The flexibility profile was based on the Back-Saver Sit and Reach test, which measures hamstring flexibility and joint range of motion. The specific measurement process of each physical fitness indicator is represented in Supplementary Table 1. More details regarding the test protocols can be found elsewhere^24,28,31,32,34,35^.

### Other studied covariates

Data describing the structural characteristics of schools were obtained from the central database of the Educational Authority Public Education Information System. The locations of the schools involved in these analyses were categorized as capital city, city, town, or village. Schools were grouped into the following categories based on their maintainer: central budgetary schools, foundations, local government, other (private person or association), and church.

The analyses also accounted for school subtypes: school centers (headquarters), which represent larger schools encompassing multiple educational levels or vocational programs, and branch schools (task execution sites), which are smaller education facilities in specific locations where educational activities occur. If an institution operates in multiple locations, each location counts as a separate task execution site, whereas a school center refers to a larger organizational unit that may include multiple sites.

A school-level indicator was included due to the longitudinal design, as student mobility and major policy reforms led to major changes in structural characteristics such as school maintainers. These changes introduced substantial year-to-year variation in school characteristics, necessitating adjustment for potential confounding effects. In the interpretation these variables handled as proxies to control for the potential confounding effects of locality (urban vs. rural) and differences in school-level resources associated with school type and maintainer, such as availability of sports facilities and teacher motivation.

### Statistical analysis

Prior to the analyses, a multistage data cleaning process was conducted, including sorting out cases with missing data or extreme values. The frequencies of the students who did not meet the healthy fitness zone (NMHFZ) were presented as point estimates and were separated by sex.

Due to the complex nature of the data, where individual student test results are paired and students are partially crossed within schools in the cohort, we used generalized linear mixed-effects models (GLMMs) to analyze individual-level trends across the studied school years. This approach accounts for the intra-individual correlation of repeated measures and the intra-school correlation of students, leading to more accurate estimates of standard errors and allowing for the modelling of both fixed effects (age, sex, school year, and structural characteristics of schools) and random effects (individual variation among students and clustering within schools) simultaneously. Our modelling approach began with the inclusion of random effects (school and student) to account for hierarchical data structure. The necessity of random effects was assessed using ANOVA to compare a simple intercept-only model with a model that included random effects. If the random effects model significantly explained more of the variance, we proceeded with it and evaluated the inclusion of fixed effects. When the data supported the use of multilevel mixed-effects models, we estimated the intraclass correlation coefficient (ICC) to quantify the proportion of variance attributable to differences between participants relative to the total variance.

Using binomial random-effect models, we investigated the influence of COVID-19 independently of demographic factors (age and sex) and school characteristics (location, maintainer, and local control) on the nine studied primary physical fitness indicators. A maximum likelihood estimator (ML) was used in the models, and 95% CIs and p-values were computed using a Wald t-distribution approximation. Multicollinearity among school-level covariates was assessed using Variance Inflation Factor (VIF). The analysis was conducted using complete-case data, with all independent variables fully observed and participants with missing values on the dependent variables excluded from the analyses. Associations were quantified by odds ratios (ORs), which represent the odds of not meeting the healthy fitness zone (NMHFZ). Based on the multivariate GLMMs, the distributions of the conditional modes were presented using caterpillar plots. The diagrams represent the log-odds of NMHFZ for each school, adjusted for fixed effects such as student demographics (sex and age) and school characteristics (location, maintainer, and local control). The curved line illustrates the estimated random intercepts for each school, along with their 95% confidence intervals, capturing school-specific deviations from the overall baseline probability of failure to meet the healthy fitness zones. Schools with intercepts above the average have a higher probability of students not meeting the healthy fitness standards than the typical school, whereas schools below the average line have a lower probability.

In the final step, as a sensitivity analysis, models were supplemented with interaction terms between adolescents’ age and sex and the studied school years to assess the potential effect modification of students’ demographic variables on the physical fitness profile change. This was considered important since changes in the fitness profile may show variation according to the sex and age of the pupils.

All statistical analyses were performed using R version 4.0.5 (2021-03-31) -- “Shake and Throw” using packages “tidyverse,” “lme4,” and “jtools”.

## Results

**Fig. 1:**
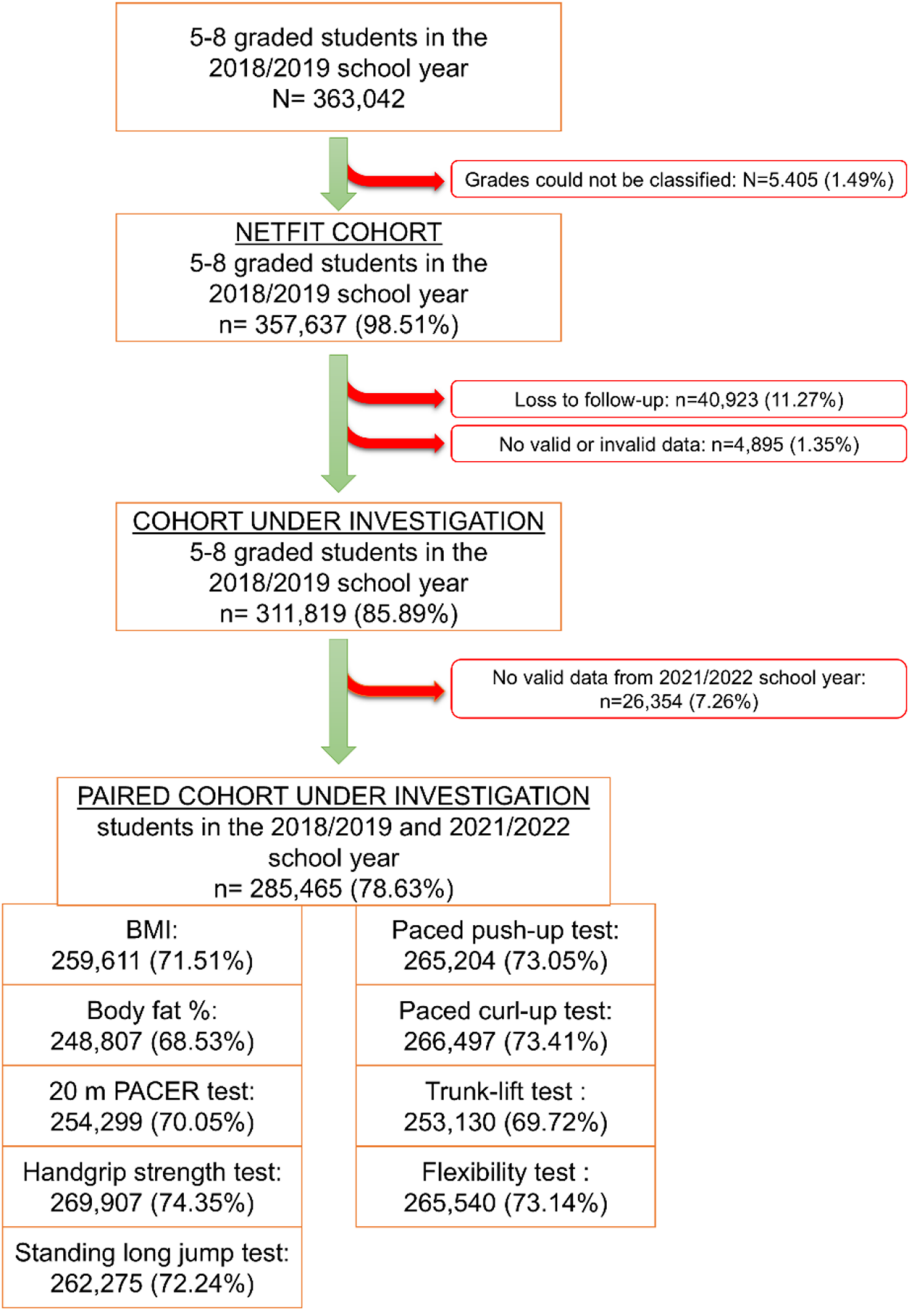
Flow diagram for the selection of participants included in the final analysis.

A total of 657,691 students participated in the NETFIT^®^ measurement in pre-pandemic, and 680,892 in post-pandemic school year. Restricting this population to students in grades 5 to 8 in the pre-pandemic school year resulted in a cohort of 357,637 students, representing 98.51% of the total target population. After excluding 40,923 students due to loss of follow-up (leaving school early), 4,895 due to a lack of data, and 26,354 due to data error or completely missing records, the final cohort was reduced to 285,465 students, representing 78.63% of the initial target population of all students in grades 5 to 8 in Hungary at the end of the two-year follow-up period (Fig. 1).

In the pre-pandemic school year, 51.19% of the students were boys, with an average age of 12.54 years (SD: ±1.26). Most schools were funded by a central budget institution (77.21%) in the pre-pandemic era, decreasing to 42.35% in post-pandemic period. Nearly 90% of the schools were school centers in pre-pandemic, compared to 60.95% in post-pandemic time. This substantial change is explained by the specificities of the Hungarian education system due to the high rate of school transition of students after the 7^th^ and 8^th^ grades. The geographical distribution remained similar, with less than a quarter (23.46%) of schools located in rural areas (Table 1).

**Table 1:** Demographic characteristics of the respondents and the school profiles of the cohort studied.

	Pre-pandemic	Post-pandemic
Sex		
Boys	146,119 (51.19%)	
Girls	139,346 (48.81%)	
Location		
Capital city	44,100 (15.45%)	55,035 (19.28%)
City	65,597 (22.98%)	101,634 (35.60%)
Town	108,805 (38.12%)	107,254 (37.57%)
Village	66,963 (23.46%)	21,542 (7.55%)
Maintainer		
Central budgetary	220,409 (77.21%)	120,893 (42.35%)
Foundations	5,162 (1.81%)	9,573 (3.35%)
Local government	7,137 (2.50%)	100,950 (35.36%)
Other (e.g.: private person, association)	1,246 (0.44%)	1,927 (0.68%)
Church	51,511 (18.04%)	52,122 (18.26%)
School subtype		
Branch	38,299 (13.42%)	111,466 (39.05%)
School center	247,166 (86.58%)	173,999 (60.95%)

Data are presented as n (%).

The percentage of children whose BMI did not meet the healthy fitness zone (NMHFZ) remained approximately the same in pre-pandemic (26.77%) and post-pandemic (26.65%) eras. However, the number of students who classified NMHFZ for body fat percentage (BF%) increased from 31.37% to 34.32%. The percentage of students with NMHFZ for handgrip strength also increased slightly, from 40.66% to 41.68%. In the post-pandemic school year, the percentage of students with NMHFZ in the standing long jump (24.37% to 28.30%) and push-up tests (28.39% to 33.40%) increased. More students were included in the NMHFZ for the trunk-lift (49.84% to 55.25%) and flexibility (33.87% to 37.76%) tests in post-pandemic era. The greatest increase was observed in the 20 m PACER test, where the NMHFZ rate increased from 32.62% to 53.38% (Table 2).

**Table 2:** Crude prevalence of students who fail to meet the healthy fitness zone by sex among the studied cohort in Hungary, according to the NETFIT^®^ measurement.

	Pre-pandemic			Post-pandemic			Missing values (pre-pandemic; post-pandemic)
	Total	Boys	Girls	Total	Boys	Girls	
BMI	*74*,*384 (26.77%)*	41,324 (29.11%)	33,060 (24.32%)	*70*,*990 (26.65%)*	40,611 (29.87%)	30,379 (23.30%)	2.66%; 6.70%
Body Fat%	*85*,*620 (31.37%)*	44,615 (32.18%)	41,005 (30.54%)	*88*,*895 (34.32%)*	44,492 (33.85%)	44,403 (34.81%)	4.39%; 9.27%
20 m PACER test	*89*,*994 (32.62%)*	43,223 (30.52%)	46,771 (34.82%)	*140*,*037 (53.38%)*	68,455 (50.30%)	71,582 (56.70%)	3.34%; 8.11%
Handgrip strength test	*114*,*138 (40.66%)*	59,333 (41.28%)	54,805 (40.01%)	*114*,*280 (41.68%)*	54,686 (38.96%)	59,594 (44.54%)	1.68%; 3.96%
Standing long jump test	*67*,*795 (24.37%)*	39,907 (27.99%)	27,888 (20.57%)	*76*,*021 (28.30%)*	46,162 (33.31%)	29,859 (22.97%)	2.56%; 5.91%
Push-up test	*79*,*412 (28.39%)*	42,624 (29.72%)	36,788 (27.00%)	*90*,*280 (33.40%)*	53,104 (38.01%)	37,176 (28.46%)	2.03%; 5.30%
Curl-up test	*23*,*366 (8.35%)*	13,018 (9.08%)	10,348 (7.58%)	*22*,*854 (8.42%)*	13,243 (9.48%)	9,611 (7.29%)	1.94%; 4.88%
Trunk-lift test	*136*,*482 (49.84%)*	64,415 (45.72%)	72,067 (54.19%)	*145*,*300 (55.25%)*	69,212 (51.06%)	76,088 (59.71%)	4.07%; 7.88%
Flexibility test	*94*,*814 (33.87%)*	52,102 (36.38%)	42,712 (31.24%)	*102*,*087 (37.76%)*	41,204 (29.79%)	60,883 (46.11%)	1.93%; 5.30%

Data are presented as n (%).

### Changes in students’ fitness profiles

Multivariate analyses revealed an increase in the proportion of students whose BMI, BF%, 20 m PACER, flexibility, push-up, and curl-up test results did not meet the healthy fitness zone in the post-pandemic school year compared with the pre-pandemic period (OR_BMI_=1.22, OR_BF%_=1.16, OR_PACER_=1.65, OR_FELXIBILITY_=1.06, OR_PUSH−UP_=1.19, and OR_CURL−UP_=1.17). Conversely, the likelihood of NMHFZ decreased for handgrip strength, standing long jump, and trunk-lift tests (OR_HANDGRIP_=0.92, OR_JUMP_=0.93, and OR_TRUNK−LIFT_=0.95) (Tables 3 and 4).

**Table 3:** Factors associated with not meeting the healthy fitness zone in body composition, endurance and flexibility profiles of Hungarian school-aged children before and after the COVID-19 pandemic (pre-pandemic and post-pandemic school years) based on the multivariate generalized linear mixed-effects models.

	BMI^a^ OR (95% CI)			Body Fat%^a^ OR (95% CI)		20 m PACER test OR (95% CI)		Flexibility test OR (95% CI)	
School year									
Post-pandemic (ref.: Pre-pandemic)	1.22 (1.14–1.31)***			1.16 (1.12–1.20)***		1.65 (1.61–1.70)***		1.06 (1.04–1.09)***	
Sex									
Female (ref.: Male)	0.67 (0.64–0.71)***			0.95 (0.93–0.98)***		1.57 (1.55–1.60)***		1.38 (1.36–1.40)***	
Age	0.87 (0.83–0.90)***			1.01 (0.99–1.03)		1.74 (1.71–1.76)***		1.05 (1.03–1.06)***	
Location									
City (ref.: Capital city)	1.26 (1.17–1.36)***			1.31 (1.23–1.41)***		1.20 (1.06–1.37)**		1.01 (0.90–1.12)	
Town (ref.: Capital city)	1.67 (1.55–1.79)***			1.67 (1.57–1.78)***		1.54 (1.36–1.73)***		1.06 (0.96–1.17)	
Village (ref.: Capital city)	1.76 (1.62–1.92)***			1.71 (1.60–1.83)***		1.75 (1.56–1.98)***		0.94 (0.85–1.04)	
Maintainer									
Foundations (ref.: Central budgetary)	0.70 (0.60–0.81)***			0.80 (0.71–0.91)***		0.85 (0.68–1.05)		0.90 (0.75–1.08)	
Local government (ref.: Central budgetary)	1.23 (1.14–1.33)***			1.34 (1.25–1.43)***		1.05 (0.93–1.20)		1.13 (1.01–1.26)*	
Other (ref.: Central budgetary)	1.45 (1.12–1.88)**			0.64 (0.51–0.80)***		0.69 (0.48–0.99)*		0.98 (0.73–1.31)	
Church (ref.: Central budgetary)	0.96 (0.90–1.02)			0.97 (0.92–1.03)		0.89 (0.80–0.98)*		0.97 (0.90–1.06)	
School subtype									
School center (ref.: Branch)	0.91 (0.86–0.97)**			0.83 (0.79–0.88)***		0.66 (0.60–0.72)***		0.85 (0.78–0.92)***	
Interaction: Sex (Female) x School year (Post-pandemic)^b^	0.70 (0.66–0.74)***			1.38 (1.33–1.43)***		1.29 (1.25–1.33)***		3.77 (3.67–3.87)***	
Interaction: Age x School year (Post-pandemic)^b^	0.89 (0.85–0.93)***			1.37 (1.33–1.41***		1.28 (1.25–1.31)***		1.39 (1.36–1.42)***	
n_school_	3,691			3,641		3,652		3,681	
n_student_	259,611			248,807		254,299		265,540	
ICC_student_	0.97			0.57		0.25		0.20	
ICC_school_	0.01			0.03		0.19		0.15	

^a^Underweight treated as healthy fitness zone, underweight classification in NETFIT^®^ is based on Cole et al.^33^

***p < 0.001, **p < 0.01, *p < 0.05.

^b^Results are based on the sensitivity analysis, where interaction terms between sex, age and school years are added to the model.

OR (95% CI): odds ratio (95% confidence interval); n_school_: number of schools investigated; n_student_: student number; ICC: intraclass correlation coefficient.

**Table 4:** Factors associated with not meeting the healthy fitness zone in the musculoskeletal fitness profile of Hungarian school-aged children before and after the COVID-19 pandemic (pre-pandemic and post-pandemic school years) based on the multivariate generalized linear mixed-effects models.

	Handgrip strength test OR (95% CI)	Standing long jump test OR (95% CI)	Push-up test OR (95% CI)	Curl-up test OR (95% CI)	Trunk-lift test OR (95% CI)
School year					
Post-pandemic (ref.: Pre-pandemic)	0.92 (0.90–0.95)***	0.93 (0.90–0.96)***	1.19 (1.17–1.22)***	1.17 (1.13–1.21)***	0.95 (0.93–0.97)***
Sex					
Female (ref.: Male)	1.10 (1.08–1.12)***	0.51 (0.50–0.52)***	0.75 (0.74–0.76)***	0.81 (0.79–0.82)***	1.52 (1.50–1.54)***
Age	1.09 (1.08–1.11)***	1.31 (1.29–1.33)***	1.13 (1.12–1.15)***	1.02 (0.99–1.04)	1.24 (1.22–1.25)***
Location					
City (ref.: Capital city)	0.85 (0.79–0.91)***	1.54 (1.38–1.72)***	1.31 (1.17–1.46)***	1.33 (1.12–1.58)***	0.77 (0.67–0.88)***
Town (ref.: Capital city)	0.73 (0.69–0.78)***	1.98 (1.79–2.20)***	1.47 (1.32–1.63)***	1.89 (1.62–2.21)***	0.84 (0.74–0.96)**
Village (ref.: Capital city)	0.62 (0.58–0.66)***	2.30 (2.07–2.56)***	1.56 (1.41–1.74)***	2.44 (2.08–2.86)***	0.92 (0.81–1.05)
Maintainer					
Foundations (ref.: Central budgetary)	1.05 (0.93–1.19)	0.83 (0.68-1.00)	0.73 (0.60–0.88)**	0.90 (0.68–1.20)	1.20 (0.95–1.52)
Local government (ref.: Central budgetary)	0.89 (0.83–0.95)**	1.01 (0.91–1.13)	0.95 (0.85–1.06)	0.79 (0.67–0.94)**	1.07 (0.94–1.23)
Other (ref.: Central budgetary)	1.41 (1.14–1.74)**	0.78 (0.57–1.08)	0.53 (0.39–0.72)***	1.22 (0.78–1.89)	1.12 (0.78–1.60)
Church (ref.: Central budgetary)	0.95 (0.90-1.00)	0.87 (0.80–0.95)**	0.87 (0.80–0.95)**	0.75 (0.66–0.86)***	1.09 (0.98–1.20)
School subtype					
School center (ref.: Branch)	0.97 (0.92–1.02)	0.66 (0.60–0.71)***	0.81 (0.74–0.87)***	0.62 (0.55–0.70)***	1.07 (0.97–1.18)
Interaction: Sex (Female) x School year (Post-pandemic)^a^	1.41 (1.37–1.44)***	0.87 (0.85–0.90)***	0.72 (0.70–0.74)***	1.00 (0.95–1.04)	0.95 (0.92–0.97)***
Interaction: Age x School year (Post-pandemic)^a^	1.27 (1.24–1.30)***	1.31 (1.28–1.35)***	0.87 (0.85–0.89)***	1.06 (1.02–1.10)**	0.86 (0.85–0.88)***
n_school_	3,688	3,674	3,697	3,691	3,692
n_student_	269,907	262,275	265,204	266,497	253,130
ICC_Student_	0.34	0.36	0.21	0.09	0.07
ICC_School_	0.05	0.12	0.16	0.33	0.26

***p < 0.001, **p < 0.01, *p < 0.05.

^**a**^Results are based on the sensitivity analysis, where interaction terms between sex, age and school years are added to the model.

OR (95% CI): odds ratio (95% confidence interval); n_school_: number of schools investigated; n_student_: student number; ICC: intraclass correlation coefficient.

Female students had lower odds of NMHFZ for BMI (OR = 0.67), BF% (OR = 0.95), standing long jump (OR = 0.51), push-up (OR = 0.75), and curl-up tests (OR = 0.81). However, they were more likely to fall outside the HFZ in the 20 m PACER (OR = 1.57), flexibility (OR = 1.38), handgrip strength (OR = 1.10), and trunk-lift tests (OR = 1.52). Student age was associated with lower odds of NMHFZ for BMI (OR_BMI_= 0.87). However, increasing age was linked to higher odds of NMHFZ for the 20 m PACER (OR = 1.74), flexibility (OR = 1.05), handgrip strength (OR = 1.09), standing long jump (OR = 1.31), trunk-lift (OR = 1.24), and push-up tests (OR = 1.13) (Tables 3 and 4).

Compared with those in capital city, students in cities, towns, and villages were more likely to be NMHFZ for the BMI, BF%, 20 m PACER, standing long jump, push-up, and curl-up tests.

A graded pattern was observed, with progressively higher odds of NMHFZ from cities to towns and villages. Among students living in cities, the odds ratios ranged from 1.20 for the 20 m PACER to 1.54 for the standing long jump. Higher odds were observed among students from towns (OR_BMI_ = 1.67; OR_BF%_ = 1.67; OR_PACER_ = 1.54; OR_JUMP_ = 1.98; OR_PUSH−UP_ = 1.47; OR_CURL−UP_ = 1.89), and villages (OR_BMI_ = 1.76; OR_BF%_ = 1.71; OR_PACER_ = 1.75; OR_JUMP_ = 2.30; OR_PUSH−UP_ = 1.56; OR_CURL−UP_ = 2.44).

Conversely, the odds of NMHFZ for handgrip strength were lower among students in cities (OR = 0.85), towns (OR = 0.73), and villages (OR = 0.62). A similar trend was observed for the trunk‒lift test, where students in cities (OR = 0.77) and towns (OR = 0.84) had lower odds of NMHFZ (Tables 3 and 4).

Students in foundational schools were less likely to be NMHFZ for the BMI, BF% and push-up tests (OR_BMI_=0.70, OR_BF%_=0.80, and OR_PUSH−UP_= 0.73). Those attending church-maintained schools had lower odds of NMHFZ in PACER (OR = 0.89), standing long jump (OR = 0.87), push-up (OR = 0.87), and curl-up (OR = 0.75) tests. Similarly, students in local government-maintained schools had a reduced likelihood of poor performance in handgrip strength (OR = 0.89) and curl-up (OR = 0.79) tests. Conversely, students in local government-maintained schools were more likely to be NMHFZ for BMI, BF%, and flexibility tests (OR_BMI_=1.23, OR_BF%_=1.34, and OR_FELXIBILITY_=1.13) (Tables 3 and 4**).**

Additionally, students in school centers had more favorable test results for BMI (OR = 0.91), BF% (OR = 0.83), 20 m PACER (OR = 0.66), flexibility (OR = 0.85), standing long jump (OR = 0.66), push-up (OR = 0.81), and curl-up (OR = 0.62) than their counterparts in branch schools did (Tables 3 and 4).

### School-level variation between fitness profiles of the students

**Fig. 2:**
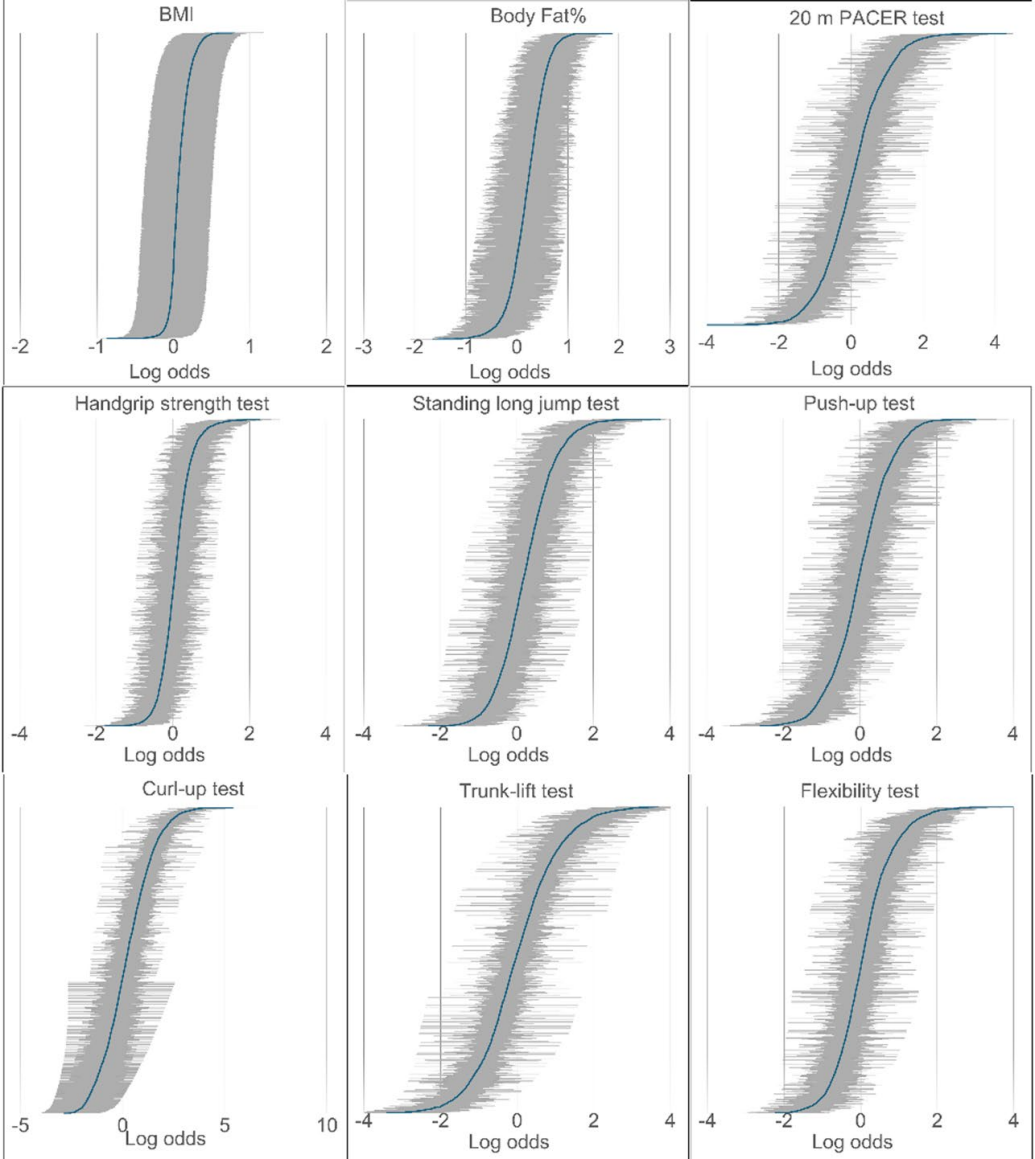
School-level variation in fitness profiles of the students: random intercepts from a mixed-effects logistic regression model represent the log-odds of not meeting the healthy fitness zone.

Figure 2 illustrates the variation in fitness profiles across schools. This allows us to visually compare differences in student fitness outcomes at the school level while accounting for demographic and school-level factors. A log-odds of 0 represents the overall average for school-level performance. Log-odds above 0 indicate a higher probability of students not meeting healthy fitness standards, whereas below 0 indicate a lower probability. Compared with the BMI, BF%, and handgrip strength tests, the 20 m PACER, flexibility, standing long jump, curl-up, and trunk-lift tests exhibit a wider spread of random intercepts, suggesting significant differences in the school-specific probabilities of the NMHFZ standards. Most schools perform close to the overall average on fitness tests, with few extreme differences.

Greater between-school variability was observed in the 20 m PACER test, with approximately one-quarter of schools performing above and one-quarter below the average. Variability was detected in the trunk-lift test, where about one-third of schools were classified as both above and below average. In addition, a considerable proportion of schools demonstrated below-average performance in muscular fitness tests, particularly the standing long jump (31% below average), push-up (25% below average), and curl-up (34% below average). (Table 5**).**

**Table 5:** School-level variation in student fitness profiles: proportion of schools significantly deviating from average log-odds of not meeting the health fitness zone in generalized linear mixed-effects models.

	Under average	Approximately average	Above average
BMI	11 (0.30%)	3,608 (97.75%)	72 (1.95%)
Body Fat%	106 (2.91%)	2,805 (77.04%)	730 (20.05%)
20 m PACER test	932 (25.52%)	1,785 (48.88%)	935 (25.60%)
Handgrip strength test	293 (7.94%)	2,842 (77.06%)	553 (14.99%)
Standing long jump test	410 (11.16%)	2,108 (57.38%)	1,156 (31.46%)
Push-up test	890 (24.07%)	1,880 (50.85%)	927 (25.07%)
Curl-up test	473 (12.81%)	1,973 (53.45%)	1,245 (33.73%)
Trunk-lift test	1,181 (31.99%)	1,384 (37.49%)	1,127 (30.53%)
Flexibility test	770 (20.92%)	1,978 (53.74%)	933 (25.35%)

Data are presented as n (%).

### Sensitivity analyses

The interactions between sex and school years suggested that, during the analyzed period, girls presented greater odds of NMHFZ for BF% (OR = 1.38), 20 m PACER test (OR = 1.29), flexibility (OR = 3.77) and handgrip strength (OR = 1.41) tests, whereas BMI (OR = 0.70), standing long jump (OR = 0.87), push-up (OR = 0.72) and trunk-lift (OR = 0.95) remained relatively stable among girls (Tables 3 and 4). Within the study period, the deterioration was more pronounced among older students in terms of the BF% (OR = 1.37), 20 m PACER (OR = 1.28), flexibility (OR = 1.39), handgrip strength (OR = 1.27), standing long jump (OR = 1.31) and curl-up (OR = 1.06) tests, while younger students were more likely to have lower BMI (OR = 0.89) and push-up (OR = 0.87) test results (Tables 3 and 4).

## Discussion

We examined a cohort of over 250,000 youth from grades 5 to 8 over two years to assess changes in various aspects of physical fitness in response to COVID-19 restrictions, controlling for student age, sex and school characteristics. Our findings indicate a decline in body composition, cardiorespiratory fitness, and flexibility. Interestingly, musculoskeletal fitness showed mixed results; while performance in the push-up and curl-up tests deteriorated, measures such as handgrip strength, standing long jump, and trunk-lift test demonstrated improvement. Notably, the decline in body composition, cardiorespiratory fitness, and muscle endurance (push-up and curl-up tests) was significantly greater than the gains observed in case of handgrip, standing long jump and trunk-lift tests. This suggests non-uniform changes in students’ fitness profiles during the COVID-19 restrictions, which varied by the domain being investigated.

The body composition and cardiorespiratory fitness changes demonstrated in this study are consistent with previous research that has shown that prolonged periods of inactivity can have negative effects on different fitness profiles^10,12,13,15,18,36–38^. Furthermore, many investigations have demonstrated a decline in cardiovascular endurance^10,13,15,16,37,38^, highlighting the broader impact of restricted physical activity during this period.

There is no clear consensus regarding how lockdown correlated adolescents’ musculoskeletal systems. Similar to our study, studies from Estonia, the USA, and Poland^16,39^ reported decreases in push-up and curl-up test results after the COVID-19 pandemic. However, handgrip strength, standing long jump, and trunk-lift scores improved in Hungary and elsewhere^10,16,40^. We explain these results obtained by handgrip strength, standing long jump, and trunk-lift tests are primarily used to assess maximal strength and explosive power, whereas push-ups and curl-ups require muscular endurance, which is more susceptible to decline when physical activity levels decrease. The flexibility results were also mixed. Chinese^36,38^ studies reported gains, whereas Greek^40^ and French^14^ studies reported decreases. As our methods align more with European studies, our findings support their results. Overall, unlike body composition and cardiovascular fitness, to the effects of the lockdown on musculoskeletal test results were selective. Strength and power were maintained or improved, whereas endurance decreased due to less activity and structure.

In addition to the clear sex and age differences identified by statistical models which may be related tophysiological, hormonal, and behavioral factors, sensitivity analysis revealed that sex and age modified the effects of COVID-19 closures. Lockdown impacts were stronger in girls for BF%, cardiovascular endurance, flexibility, and handgrip strength, whereas boys showed greater changes in BMI, standing long jump, push-up, curl-up, and trunk-lift. Age also exerted an effect modification. Younger students had greater increases in BMI and declines in push-up performance, whereas older students showed greater declines in BF%, cardiovascular endurance, flexibility, and curl-ups. Older students also improved more in handgrip strength and long jump, whereas trunk-lift gains were greater among younger students. Our study, like others, revealed worsened body composition and improved cardiovascular endurance in boys^11,12,38^. Boys’ greater weight gain and girls’ sharper performance decline are likely due to reduced activity, more sedentary time, and mismatched energy intake, especially in boys. Boys’ greater muscle mass and strength may have supported their performance levels.

Similar to other studies^12,41^, considering age as an effect modification factor was linked to variations in the fitness profile of the students, especially among older adolescents. Several facilitating factors may contribute to these results. Compared with their older peers, young students are more likely to participate in free play and unstructured physical activity during leisure time (such as biking)^8^, in addition, studies have shown that older students tend to move less^42,43^. The results of the body composition profile are most negatively affected by age. This underlines the importance of fostering joy for movement as early as possible so that regular physical activity may become a part of children’s daily routine.

To the best of our knowledge, this is the first study to analyze not only the impact of COVID-19 on students’ physical fitness profiles, but also the structural characteristics of educational institutions. The analysis revealed substantial variation at the school level. For BF%, the standing long jump test, and the curl-up test, a significant number of schools deviated from the average, shifting towards positive log-odds, which suggests a greater likelihood of unfavorable test outcomes in many schools. The 20 m PACER, flexibility, curl-up, and trunk-lift tests exhibited the greatest variability across schools, with both favorable and unfavorable results.

The settlement size also mattered. Compared with those in the capital, students in smaller towns were less likely to reach the HFZ for body composition, cardiorespiratory fitness, and musculoskeletal fitness. This likely may be related to Hungary-specific factors, including limited sports infrastructure and programs in smaller or rural areas. Additionally, local attitudes and weak leisure policies often reduce engagement in physical activity. In contrast, in rural communities, there is often a negative perception of local opportunities and the leisure time policy actions of local governments^44^, which may contribute to less popular community leisure activities.

School maintainer type also related the investigated outcomes in this study. Students in foundation, church, or central schools were more likely to meet HFZ standards. These schools may promote healthier lifestyles by integrating physical, mental, and spiritual development. They also tend to have better resources and more structured activities and may attract students from higher socioeconomic backgrounds. However, it is possible that these differences partly reflect underlying socioeconomic or demographic characteristics rather than being solely attributable to school type. Additionally, we hypothesized that students attending school centers benefit from better-trained physical education teachers, a wider variety of extracurricular sports programs, more structured physical activity opportunities, and greater access to high-quality facilities.

A key strength of the present investigation is the ability to test changes in students’ physical fitness profiles over time using prospective, cohort data with a relatively small dropout rate (11%) from the Hungarian adolescent population, which allowed for a thorough assessment of observed changes in students’ physical fitness during the COVID-19 period. All of the studied outcomes were validated and objectively measured each year, which increased the reliability and validity of the data. The data collection system on which the analysis is based has been in place for a long time; therefore, the teachers have considerable experience collecting the data, which improves the data quality of the investigation. Factors such as the socioeconomic status and health behaviors of the students may provide further insights into the observed differences, and the paired study design effectively minimizes the variability caused by individual differences, thereby enhancing the study’s reliability.

However, several limitations should be acknowledged. First, we cannot determine and analyze the causes of the observed changes in physical fitness due to factors (diet, sleep, and sedentary behavior or psychological effects, including stress, anxiety, and depression) that developed among the students during the COVID-19 pandemic and the closures. Evidence from the literature^45^ suggests that key health behaviors—sleep, and diet—worsened following COVID-19. Poor diet quality and insufficient sleep can reduce energy, hinder recovery, and impair overall physical performance. Second, the study did not examine the mediating relationships between demographic factors and the impact of lockdown, which could provide valuable insights into the research. A further limitation of our study is that physical fitness data were collected at different time points across the two analysed periods. In the pre-pandemic, measurements were limited to a 19-week window (January to June), whereas in the post-pandemic data could be registered throughout the school year (September to June). Because the exact timing of individual measurements could not be analysed or controlled, we cannot rule out the possibility that seasonal variations in physical activity contributed to the observed differences in fitness outcomes. Measurements during colder or less active periods might underestimate these outcomes. Because long-term trends were not analysed, pandemic-related effects cannot be fully separated from ongoing secular changes; nevertheless, national reports indicate that pre-COVID year-to-year variation was minimal. Although minor differences were observed between students retained in the cohort and those lost to follow-up, the magnitude of these differences was small and unlikely to meaningfully bias the results. Given the high follow-up rate (89%), the possibility of residual attrition bias cannot be entirely excluded and should be considered when interpreting the findings. Nonlinear effects of age were not explicitly modeled, and age was included primarily to control for confounding; future studies could explore potential nonlinear age trends.

## Conclusion

This study offers valuable insights into adolescents’ physical fitness profiles, serving as a critical tool for health monitoring and policy development. We demonstrated that physical fitness outcomes among school-aged children changed heterogeneously during the studied period. While poorer performance was observed in several fitness components, the magnitude and direction of change were not uniform across all tests. Most notably, cardiorespiratory fitness showed a marked decline, with nearly a 20% higher probability of worsening in aerobic fitness indicators. Nevertheless, the public health relevance of smaller effect sizes warrants careful interpretation. Given their modest magnitude, some observed differences may be transient and may already have attenuated—an issue that cannot be analyzed within investigation and would require long-term follow-up data. From an intervention and policy perspective, prioritization is therefore essential: the more consistent and substantial associations observed for BMI, BF%, cardiorespiratory fitness, push-up, and curl-up performance suggest that these domains should represent primary targets for remedial strategies.

Unlike previous surveys, our investigation highlights school-level factors that may contribute to variations in students’ physical performance, with correlations likely persisting beyond the context of COVID-19. These findings underscore the need for targeted, context-sensitive interventions addressing both individual and school-level determinants. Although the lack of long-term follow-up and potential unmeasured confounders limit causal inference, the results can guide policymakers in prioritizing fitness components for intervention and pinpointing areas where schools and educators require additional support, both during public health emergencies and routine education. Further research is needed to explore the deeper influence of school structural characteristics on students’ physical performance.

## Supplementary Information

Below is the link to the electronic supplementary material.

Supplementary Material 1

## Data Availability

The datasets used and/or analyzed during the current study are available from the corresponding author on reasonable request.
