# Supplementary material for: Physical fitness changes among school-aged children during the COVID-19 lockdown evaluated within the Hungarian National Student Fitness Test cohort
Source: Sci Rep. 2026 Feb 23;16:10254. doi: 10.1038/s41598-026-41055-8 (PMC13031300; doi:10.1038/s41598-026-41055-8)
Supplement: Supplementary file 1 — Supplementary Material 1 [file 41598_2026_41055_MOESM1_ESM.docx]

# **Supplementary**

**Supplementary Table 1:** *Definitions of tests used for measurements*

| **Measurement** | **Rationale** | **Definition** |
| --- | --- | --- |
| Progressive Aerobic Cardiovascular Endurance test (PACER) | Measure the student's aerobic capacity. Higher distances indicating better aerobic capacity. | The pace increases every minute, up to 21 stages or until the participant reaches voluntary exhaustion. The test ends if a participant twice fails to reach the 20 m lap within the allotted time. The total laps completed are used to estimate VO₂ peak using the latest Fitnessgram prediction equation. |
| Flexibility test (Back-Saver Sit and Reach test) | Measure the student's hamstring flexibility and joint range of motion. A healthy test result indicates adequate hamstring flexibility for optimal fitness. | The test uses a measuring box, with the participant seated, feet flat against the box, and shoes removed. One leg remains straight while the other is bent. With arms extended and hands stacked, the participant reaches forward as far as possible while keeping a straight back. The test is repeated with the opposite leg, and the average of both measurements determines the score. |
| Standing broad jump test | Measures the student's leg strength and power. A result within the healthy range indicates sufficient leg strength for optimal fitness. | The standing broad jump test was conducted on a hard surface. Participants began with both feet on the starting line, swinging their arms for momentum before jumping forward. The jump had to be performed with both feet for take-off and landing. The distance from the starting line to the nearest heel was measured in centimetres. If participants landed with their hands behind their feet, they were given one retry. Each participant completed two trials, the best score registered. |
| Push-up test | Measures the student's upper body strength and endurance. A healthy test result indicates sufficient strength for optimal fitness. | Participants perform a series of push-ups, determined by the protocol and guided by sound signals. The objective is to perform as many push-ups as possible in time to the rhythm. |
| Curl-up test | Measures the student's abdominal strength and endurance. A healthy test result indicates sufficient strength for optimal fitness. | Participants perform a series of curl-ups, determined by the protocol and guided by sound signals. The objective is to perform as many curl-ups as possible in time to the rhythm. |
| Trunk-lift test | Measures the student's trunk extensor strength and flexibility. All result in the healthy fitness zone indicate sufficient trunk strength and flexibility to support spinal health. | The students lie face down on a firm surface with their forehead, feet, and hands positioned under the thighs. They lift their upper body while looking down. The goal is to lift up a maximum of 30 cm with proper form. |
| Handgrip strength test | Measures the student's forearm and hand strength. The test results reflects overall muscular strength and reflects on the density of bones. | The test uses a certified electronic dynamometer. The participant stands with feet shoulder-width apart, holding the device in their dominant hand with the arm lowered. They squeeze the grip as hard as possible. |
